# Potential Roles of Hypoxia-Inducible Factor-1 in Alzheimer’s Disease: Beneficial or Detrimental?

**DOI:** 10.3390/antiox13111378

**Published:** 2024-11-11

**Authors:** Tsu-Kung Lin, Chi-Ren Huang, Kai-Jung Lin, Yi-Heng Hsieh, Shang-Der Chen, Yi-Chun Lin, A-Ching Chao, Ding-I Yang

**Affiliations:** 1 Department of Neurology, Kaohsiung Chang Gung Memorial Hospital, Kaohsiung 833401, Taiwan; tklin@adm.cgmh.org.tw (T.-K.L.); suika68@cgmh.org.tw (C.-R.H.); chensd@adm.cgmh.org.tw (S.-D.C.); 2 College of Medicine, Chang Gung University, Taoyuan 333323, Taiwan; 3 Department of Family Medicine, National Taiwan University Hospital, Taipei 100225, Taiwan; b101101092@tmu.edu.tw; 4 Institute of Brain Science, National Yang Ming Chiao Tung University, Taipei 112304, Taiwan; h00397@nycu.edu.tw; 5 Department of Neurology, Taipei City Hospital Renai Branch, Taipei 106243, Taiwan; dab16@tpech.gov.tw; 6 Department of Neurology, Kaohsiung Medical University Hospital, Kaohsiung 807377, Taiwan; 7 Department of Neurology, College of Medicine, Kaohsiung Medical University, Kaohsiung 807378, Taiwan; 8 Department of Sports Medicine, College of Medicine, Kaohsiung Medical University, Kaohsiung 807378, Taiwan; 9 Brain Research Center, National Yang Ming Chiao Tung University, Taipei 112304, Taiwan

**Keywords:** amyloid-beta peptide (Aβ), amyloid precursor protein (APP), microglia, neurofibrillary tangle (NFT), neuroinflammation, oxidative stress, reactive oxygen species (ROS), secretase, tau hyperphosphorylation

## Abstract

The major pathological characteristics of Alzheimer’s disease (AD) include senile plaques and neurofibrillary tangles (NFTs), which are mainly composed of aggregated amyloid-beta (Aβ) peptide and hyperphosphorylated tau protein, respectively. The excessive production of reactive oxygen species (ROS) and neuroinflammation are crucial contributing factors to the pathological mechanisms of AD. Hypoxia-inducible factor-1 (HIF-1) is a transcription factor critical for tissue adaption to low-oxygen tension. Growing evidence has suggested HIF-1 as a potential therapeutic target for AD; conversely, other experimental findings indicate that HIF-1 induction contributes to AD pathogenesis. These previous findings thus point to the complex, even contradictory, roles of HIF-1 in AD. In this review, we first introduce the general pathogenic mechanisms of AD as well as the potential pathophysiological roles of HIF-1 in cancer, immunity, and oxidative stress. Based on current experimental evidence in the literature, we then discuss the possible beneficial as well as detrimental mechanisms of HIF-1 in AD; these sections also include the summaries of multiple chemical reagents and proteins that have been shown to exert beneficial effects in AD via either the induction or inhibition of HIF-1.

## 1. Pathophysiology of Alzheimer’s Disease

Alzheimer’s disease (AD) is the most common age-related neurodegenerative disease and presents with progressive memory impairment, declined cognition, and behavioral dysfunctions. Currently no effective treatment for the deteriorating clinical course of dementia is available, thus contributing substantial economic burdens to the patients’ families and eventually to the whole society [1,2,3]. According to the World Health Organization (WHO), around 55 million people suffer from dementia worldwide, with approximately 10 million cases added each year. These situations underscore dementia as one of the leading causes of mental and/or physical disability. Among various causes, AD may comprise 60–70% of dementia cases (https://www.who.int/news-room/fact-sheets/detail/dementia, accessed on 15 March 2023). Based on the report “2023 Alzheimer’s disease facts and figures” [4], nearly 6.7 million Americans aged 65 years and above are estimated to suffer from AD. By 2060, the number of AD patients, with a rather steep rise, may increase to 13.8 million. Comparing the death tolls from 2000 to 2019 among various diseases, death caused by human immunodeficiency virus, cardiac diseases, and cerebrovascular disorders declined while those from AD increased by more than 145% [5]. The total expenses for AD healthcare in 2023 were around USD 345 billion and are predicted to escalate to more than USD 1 trillion by 2050 due to the aging population [4,5].

**Figure 1:**
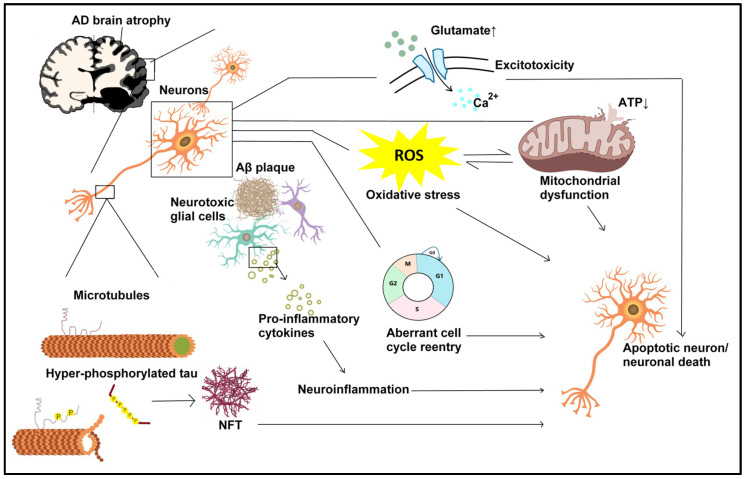
The major pathologies in AD brains include deposition of extracellular Aβ plaques and intraneuronal neurofibrillary tangles (NFTs) mainly composed of hyperphosphorylated tau proteins. Excessive Aβ aggregation can trigger diverse mechanisms including excitotoxicity, oxidative stress with heightened ROS levels, mitochondrial dysfunction with compromised ATP production, aberrant cell cycle reentry with subsequent apoptosis, and activation of neurotoxic glial cells like microglia to trigger neuroinflammation; these effects together lead to the damage or even demise of the neurons. Tau belongs to the microtubule-associated protein (MAP) family that is vital for microtubule assembly and stabilization in neuronal axons. Hyperphosphorylated tau proteins not only compromise microtubule structures to disturb axonal transport but also aberrantly aggregate into NFTs, which also contribute to neuroinflammation and neuronal apoptosis. Excessive neuronal death ultimately results in brain atrophy in AD patients.

The major pathological features in AD include atrophy of the brain [6], deposition of amyloid-beta (Aβ) plaques, and the presence of neurofibrillary tangles (NFTs) in axonal microtubules [7,8,9] (Figure 1). Aβ is a neurotoxic peptide fragment of 39–43 amino acids derived from the sequential cleavage of amyloid precursor protein (APP) by a group of enzymes called secretases [10,11]. In non-amyloidogenic pathways, most of the APP undergoes cleavage by α-secretase to yield the extracellular soluble APP-α fragment (sAPPα) and C-terminal fragment-α (α-CTF or C83); the latter is then subjected to γ-secretase-mediated proteolysis to produce p3 and an APP intracellular domain (AICD) fragment [12]. Alternatively, in the amyloidogenic pathway, a minor population of APP may first undergo β-secretase cleavage to generate sAPPβ (β-CTF or C99), followed by γ-secretase-mediated cleavage to produce Aβ and AICD [12]. Excessive Aβ aggregation can exert neurotoxicity through diverse mechanisms including excitotoxicity [13], oxidative stress [14], aberrant cell cycle reentry [15,16], mitochondrial dysfunction [17], and impaired DNA function [18], together contributing to the damage or even demise of the neurons.

Tau, a member of the microtubule-associated protein family crucial for microtubule assembly and stabilization, is involved in axonal transport in neurons [19]. The hyperphosphorylation of tau proteins not only impairs the biological functions, with resultant compromised microtubule structures and disturbed axonal transport, but also causes aberrant aggregation leading to neuronal damage [19]. Moreover, emerging evidence indicates that Aβ may also impair axonal transport and further aggravate the AD pathophysiology [20]. Additionally, Aβ can affect gene transcription and alter protein expression that may change the fate of neuronal survival or death under AD-related pathophysiological conditions [17]. Thus, any means to delay or lessen the consequences from AD may mitigate the socioeconomic impacts on the patients’ families and societies.

## 2. The Pathophysiological Roles of HIF-1 in Cancer, Immunity, and Oxidative Stress

In mammalian tissues, three hypoxia-inducible factors (HIFs) including HIF-1, HIF-2, and HIF-3 have been reported, whose expression levels are strictly regulated by oxygen tension [21]. The activation of HIF-1 is crucial for numerous cellular responses to adapt the tissues to hypoxic environments, such as increasing cell proliferation, accelerating the formation of new blood vessels, regulating glucose and energy metabolisms, affecting the synthesis of glycogen and fatty acids, and adjusting the pH level [22,23,24]. Besides HIF-1, HIF-2 is expressed in a cell-specific manner that plays a pivotal role in pulmonary development, vascularization, and erythropoiesis [25,26]. While HIF-1 and HIF-2 may function as transcription factors to drive the expression of either distinctive or overlapping target genes, the function of HIF-3 is less well understood [27]. In the present article, we only focus on the potential roles of HIF-1 in AD.

HIF-1 consists of an alpha subunit (HIF-1α) that senses the low-oxygen tension for its stabilization and a beta subunit (HIF-1β) that is constitutively expressed [28,29]. The half-life of HIF-1α in living cells under normoxic condition is only minutes [28]. This rapid degradation of HIF-1α occurs through oxygen-dependent hydroxylation catalyzed by the proteins of the prolyl hydroxylase domain (PHD) family; the hydroxylation of HIF-1α promotes binding to an E3 ubiquitin ligase, which contains the von Hippel–Lindau (VHL) protein, to facilitate its polyubiquitination and subsequent proteasomal degradation [30,31]. Under hypoxic conditions, the hydroxylation of HIF-1α is hindered due to insufficient oxygen supply, thus leading to the stabilization and accumulation of the HIF-1α subunit within the cytoplasm of a cell. In addition to hypoxic conditions, HIF-1α can also be induced by various chemical compounds such as pravastatin, deferoxamine (DFO), and cobalt chloride in miscellaneous brain cells including endothelial cells, glioma cells, and neurons under normoxic conditions [32,33,34].

**Figure 2:**
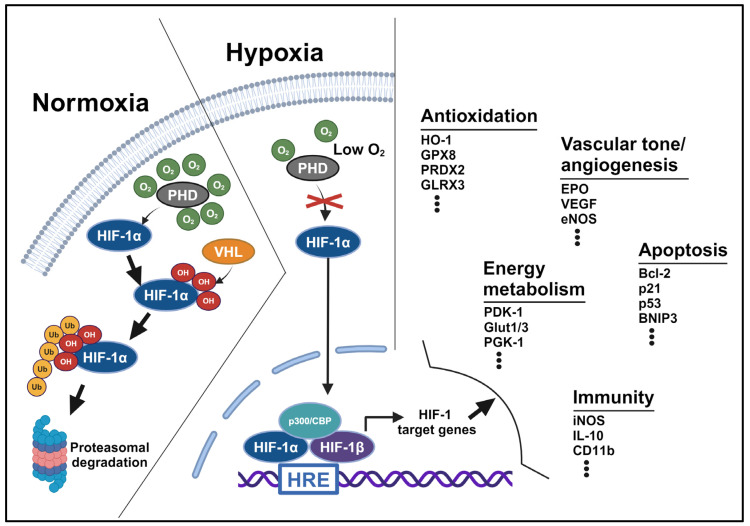
Activation of HIF-1 and its biological functions. HIF-1 is a heterodimeric transcription factor consisting of an oxygen-sensitive alpha subunit (HIF-1α) and a constitutively expressed beta subunit (HIF-1β). Under normoxia, HIF-1α undergoes hydroxylation of the proline residues catalyzed by the prolyl hydroxylase (PHD), which requires molecular oxygen (O_2_). The hydroxylated HIF-1α is then recognized by the von Hippel–Lindau (VHL) protein and E3 ubiquitin ligase for ubiquitination and subsequent proteasomal degradation. Under hypoxia, low-oxygen tension interferes with PHD hydroxylation and disrupts the interaction between HIF-1α and VHL, thereby stabilizing HIF-1α for its accumulation to form the heterodimeric HIF-1α/β. Translocation of the HIF-1α/β complex into the nucleus, along with coactivators p300/CBP, then drive the expression of target genes containing the hypoxia-response element (HRE) sequences in their promoters. HIF-1-dependent gene expression is crucial for numerous cellular responses to adapt the tissues to hypoxic environments, such as promoting angiogenesis and regulating vascular tone, enhancing antioxidation, regulating glucose transport and reprogramming energy metabolisms, affecting apoptosis, and regulating immune responses.

HIF-1α and HIF-1β each contain the structure of basic helix-loop-helix-PAS (bHLH-PAS) domains that are crucial for heterodimerization and DNA binding [28,35]. During hypoxia, translocation of the heterodimeric HIF-1α/β into the nucleus then drives the expression of target genes with the hypoxia-response elements (HREs) in their promoters [36]. Using genome-wide chromatin immunoprecipitation, hundreds of genes have been identified to be altered, either with increased or decreased expression levels, in an HIF-1-dependent manner in response to hypoxia [37]. Several well-known HIF-1 target genes including *EPO*, *VEGF*, and *PDK1*, which encode erythropoietin (EPO), vascular endothelial growth factor (VEGF), and pyruvate dehydrogenase kinase-1 (PDK1), are critical for the production of red blood cells, formation of blood vessels, and oxidative phosphorylation in the mitochondria, respectively. The heightened expression of HIF-1 target genes may assist these cells with inadequate oxygen supply in accommodating the critical hypoxic condition [23] (Figure 2).

### 2.1. Roles of HIF in Cancer

Mounting evidence supports the pivotal role of HIF-1 in tumor development, invasion, and metastasis [38]. The main components of the tumor microenvironment include blood vessels, lymphatic vessels, extracellular matrix, various immune cells, and fibroblasts [39]. A hypoxic tumor microenvironment is a driving force for cancer invasion [40,41,42]. The unmet demand for oxygen in human cancers can modify cellular metabolisms; these include the shift from oxidative phosphorylation to glycolysis, the enhanced synthesis of glycogen, and changes in the major substrate from glucose to glutamine for fatty acid synthesis. In parallel with these effects of metabolic reprogramming, HIF-1 activation may drive the expression of various genes to accommodate the hypoxic conditions and serve as a network hub to coordinate relevant signaling molecules for the promotion of tumorigenesis [24,43]. In recent years, combining HIF inhibitors with current cancer therapies to enhance anti-tumor activity is one strategy for combating therapeutic resistance [44]. This leads inevitably to an important question as to whether HIF inhibitors used in cancer patients may worsen their cognitive functions, especially in the elderly population, as age is a common risk factor for cancer and neurodegeneration. Similar concerns also exist for the clinical application of HIF inhibitors in the treatment of renal anemia [45], because the US FDA has recently approved Daprodustat, the first HIF prolyl hydroxylase (HIF-PH) inhibitor capable of blocking HIF-1 degradation, to treat anemia caused by chronic kidney disease (https://www.fda.gov/news-events/press-announcements/fda-approves-first-oral-treatment-anemia-caused-chronic-kidney-disease-adults-dialysis, accessed on 1 February 2023). Currently less well understood, these are pivotal issues that also deserve attention besides the roles of HIF-1 in AD.

### 2.2. Roles of HIF in Immunity

The immune system includes innate and adaptive components that can function efficiently through various immune-related cells such as dendritic cells, macrophages, neutrophils, B cells, and T cells. Infected and inflammatory tissues often appear with hypoxia with HIF-1 activation for adapting to hypoxic conditions; HIF-1 may also stabilize immunity under normoxic condition by regulating the metabolism and stimulating the expression of immune genes [46]. HIF-1 can affect innate immune cells in various aspects in macrophages, dendritic cells, and neutrophils. Depending on the extent of the stimuli, macrophages may polarize toward the proinflammatory status of the M1-type or toward the anti-inflammatory status of the M2-type. Notably, HIF-1α is induced in M1 macrophage polarization, whereas HIF-2α is induced during an M2 response. Macrophage polarization controls functionally divergent processes including the production of nitric oxide (NO), which is in part controlled by HIFs. Overall, NO availability is differentially regulated by HIF-1α versus HIF-2α to increase or suppress NO synthesis, respectively [47]. HIF-1α induced in lipopolysaccharide (LPS)-activated macrophages is involved in glycolysis and the induction of proinflammatory genes [48]. LPS stabilizes HIF-1α by the succinate-dependent inhibition of PHDs; further, pyruvate kinase M2 (PKM2) is also induced to promote the function of HIF-1α, which serves as a key metabolic reprogrammer for the expression of inflammatory genes [48]. Another study also reported that the glycolysis metabolism induced by HIF-1 is crucial for M1 macrophage polarization in mice [49]. Dendrite cells pivotal for antigen presenting play a significant role in innate immunity. HIF-1 is involved in the differentiation, migration, metabolism, and survival of dendrite cells under hypoxia and inflammation [50,51]. In immature dendritic cells, higher levels of HIF-1α are associated with the increased expression of pro-apoptotic molecule, such as the BCL2/adenovirus E1B 19-kDa protein-interacting protein (BNIP3) and BAX, along with the decreased expression of anti-apoptotic proteins like Bcl-2, heightened caspase-3 activity, enhanced cleavage of poly (ADP-ribose) polymerase (PARP), and cell death. On the contrary, the LPS-triggered maturation of dendritic cells attenuates hypoxia-induced cell death, wherein the PI3K/Akt pathway plays a vital part in this protective effect [51]. Pathogens may be captured and eradicated in neutrophil extracellular traps (NETs), which are mainly composed of DNA and relevant proteins generated by neutrophils. However, dysregulated NET formation may lead to excessive inflammation. A previous study showed the critical role of the mTOR/HIF-1α pathway for NET formation in neutrophils in response to LPS stimulation [52]. HIF-1 has also been shown to affect the differentiation and function of different subsets of T cells under both hypoxic and normoxic conditions. For example, HIF-1α is critical for the differentiation of various T cells including Th1, Th17, and CD8-positive effector cells; HIF-1 may also inhibit the development of regulatory T cells [53,54]. The T cell-specific knockout of HIF-1α in mice displays more severe colonic inflammation induced by dextran sodium sulfate; notably, hypoxia-induced HIF-1 is required for Treg activation but suppresses Th17 activation [55]. HIF-1α is also critical for B cell development and enhances the expression of interleukin (IL)-10 and CD11b, which may exert anti-inflammatory action [56,57,58]. B cells derived from HIF-1α-deficient mice manifested decreased IL10 and CD11b expression and became more susceptible to collagen-induced arthritis, experimental autoimmune encephalomyelitis, and inflammatory bowel diseases [58,59].

### 2.3. Roles of HIF in Oxidative Stress and Cerebral Hypoperfusion

Reactive oxygen species (ROS) are highly unstable, oxygen-containing molecules often with an unpaired electron. Fundamental for an aerobic life, the main sources of ROS in living cells include diverse origins such as the mitochondria, NADPH oxidase, cytochrome P450 enzymes, and 5-lipoxygenase [60,61,62]. To maintain the balance of redox status, cells possess the capability to counteract excessive ROS through endogenous enzymatic antioxidant systems (such as catalase, superoxide dismutase, glutathione peroxidase, and glutathione reductase) and non-enzymatic antioxidants (such as glutathione, thioredoxin, ubiquinone, and uric acid) [63,64]. However, excessive ROS may be generated under various stressful conditions such as hypoxia, inflammation, and serum deprivation [60,65]. These conditions may advance to overoxidation and result in lipid peroxidation, protein carbonylation, and DNA damage that can together proceed to cell death [66,67]. Oxidative stress is involved in numerous aging-related pathological conditions, including cancer, diabetes, chronic neurodegenerative disorders, and acute brain injuries like cerebral ischemia [68,69,70].

The role of HIF-1 in hypoxia-related oxidative stress is well-known in various ischemic conditions with inadequate oxygen supply [71,72,73,74,75]. ROS and several signal transduction pathways, including IKK/NF-kB, PI3K/AKT/mTOR, and RAS/RAF/MEK/ERK, can induce HIF-1 expression [46,76]. Downstream of HIF-1, a number of target genes involved in antioxidation with HREs in their promoters have been reported. Chromatin immunoprecipitation revealed that HIF-1α overexpression in hypoxic cardiomyocytes increases its binding to the HRE in the promoter of antioxidant gene heme oxygenase-1 (HO-1) that is known to attenuate the accumulation of ROS [77]. HO-1, but not HO-2, is also transcriptionally induced by HIF-1α in the renal medullary interstitial cells [78]. The suppression of glutathione peroxidase-8 (GPx8) enhances ER stress and cell death induced by oxidative stress in hepatocellular carcinoma cells [79]; notably, GPx8 is transcriptionally regulated by HIF-1α [80]. Peroxiredoxins (PRDXs) are a group of antioxidant enzymes that reduce hydrogen peroxide and alkyl hydroperoxides [81]; peroxiredoxin-2 (PRDX2) is a direct HIF-1 target gene whose expression is induced by prolonged hypoxia that may reciprocally interact with HIF-1α to suppress expression of other selected HIF-1 target genes under prolonged hypoxic conditions [82]. Hypoxia also affects the antioxidant activity of glutaredoxin-3 (Grx3) through HREs [83]. Depending on the experimental model systems, these previous findings together strongly support the potential antioxidative effects of HIF-1 via transcriptional induction of a multitude of antioxidation genes.

With aging, the competency for oxygen delivery in the body gradually declines and the ability to adapt hypoxia is presumed to be compromised [84,85,86]. Hypoxia may thus exacerbate the progression of neurodegenerative diseases including AD [87]. Emerging evidence has revealed that long-lasting or intermittent hypoxia, as seen in chronic obstructive pulmonary disease (COPD) or sleep apnea, is closely associated with AD [88,89]. In a prospective clinical study, older women with a sleep-related breathing disorder, which was defined as an apnea–hypopnea index of 15 or more events per hour of sleep, have an increased risk for declined cognition as compared with those without this disorder [90]. Apnea or hypopnea during sleep may therefore contribute to cognitive impairment. Nevertheless, discernible acute hypoxia is not often encountered clinically in AD patients and robust evidence for hypoxic microenvironments within the AD brains, as those seen in solid tumors, is still lacking [88,91]. With advanced neuroimaging tools, however, cerebral hypoperfusion, and accordingly hypoxia, is recognized as a constant feature along the AD continuum [92]. Evidence from neuroimaging studies showed that hypoperfusion in the brain areas, as a result of dysfunctional neurovascular units or the immunosuppressive network [93,94,95,96,97], is a potential inducer of AD pathology [98]. Cerebrovascular disease increased tau pathology in AD patients; further, transient cerebral artery occlusion as an animal model for brain hypoperfusion also promoted tau hyperphosphorylation [99]. Magnetic resonance imaging (MRI) studies showed cortical atrophy and hypoperfusion in a transgenic mouse model of AD [100]. In another human study, insufficient cerebral perfusion is correlated with cognitive deficits in AD. Regions with a lower perfusion index showed spatial similarities with atrophy in the posterior cingulate cortex, temporal lobes, and angular gyrus, while regions with lower relative cerebral blood flow were specified to the territories of distal branches in posterior cerebral artery territories [101]. It was also revealed with resting-state functional MRI that hypoperfusion may contribute to cognitive impairment via abnormal brain functional connectivity [102]. A prospective observational study in an aging population with an 11-year follow-up period has shown that anemia, wherein the compromised oxygen-carrying capability of blood may lead to brain hypoxia, was associated with an increased chance of developing dementia [103]; in another study, anemic subjects with a good baseline cognitive performance also had a two-fold higher risk of developing dementia three years later as compared to those without anemia [104]. Although the underlying mechanism linking anemia to incidental dementia is incompletely understood, one hypothesis suggested that chronic brain hypo-oxygenation is associated with an anemia-dependent risk of dementia [104]. Further studies linking cerebrovascular hypoperfusion or chronic hypoxia to dementia development are needed to further clarify these issues.

It was realized that hypoxia, oxidative stress, and neuroinflammation are crucial contributors in neurodegenerative diseases including AD [88,89]. Intriguingly, these factors may also trigger the activation of the HIF-related pathway. As compared to cancers, however, the role of HIF-1 is not well-defined in AD in terms of chronic hypoxia, oxidative stress, and inflammation, all of which are worthy of further exploration. Highly conserved during evolution, HIF-1α is stabilized under hypoxic stress so the organisms may cope with such stressful conditions. Therefore, manipulating the components of the HIF pathway may potentially exert neuroprotective effects in a variety of human neurological disorders including AD [105,106,107].

## 3. Expression Level of HIF-1α and Its Impacts on AD

**Figure 3:**
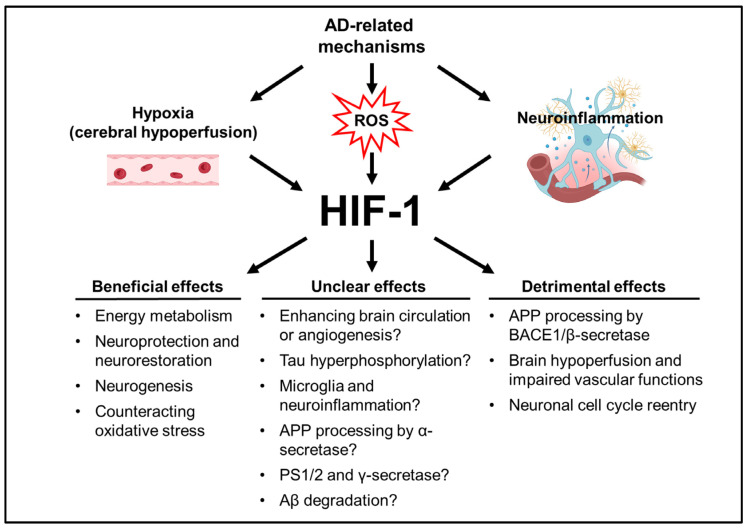
Multiple AD-related mechanisms, including cerebral hypoperfusion, oxidative stress, and neuroinflammation, may trigger activation of HIF-1 to exert either positive or negative impacts on AD progression. The beneficial effects triggered by HIF-1 include affecting energy metabolisms, promoting neuroprotection/neurorestoration, enhancing neurogenesis, and counteracting oxidative stress, together allowing the tissues to adapt to the hypoxic environment. The detrimental effects include enhancing BACE1 expression with heightened β-secretase activity to promote Aβ production, impairing brain microvascular functions, and triggering neuronal cell cycle reentry followed by apoptosis. Notably, several unclear effects of HIF-1 in AD deserve detailed investigation. These include modulating brain circulation/angiogenesis, regulating tau hyperphosphorylation, affecting microglial functions and neuroinflammation, controlling the activities of α-secretase, γ-secretase, PS1/2 functions, and even Aβ degradation.

It is well understood that the HIF system activates a wide variety of physiological responses to hypoxia, ranging from the enhancement of survival to cell cycle arrest or even cell demise [6]. Whether the Janus-faced HIF-1α acts as a “damaging factor” or a “pro-survival factor” in AD may depend on the cell types and local cellular conditions [108,109]. The altered expression of HIF-1α has been noted in AD patients as well as in transgenic AD mouse models. The downregulation of HIF-1α, which regulates the two major brain glucose transporters GLUT1 and GLUT3 for glucose uptake into neurons, was observed in AD brains; this decrease in HIF-1/GLUT1/3 correlated to the hyperphosphorylated tau and higher density of NFTs [110]. In contrast, HIF-1α is overexpressed in brain microvessels derived from AD patients [111]. Consistently in the brains of adult (50–60 weeks of age) Tg2576 AD mice, immunofluorescence staining of brain microvessels revealed a significantly higher level of HIF-1α along with the heightened expression of pro-angiogenic factors and reduced anti-apoptotic Bcl-xL [112]. Triple (3×-Tg AD; PS1M146V, APPswe, tauP301L) transgenic AD mouse brains also showed hypoxic vessels expressing HIF-1α, which resulted in the formation of NLR family pyrin domain-containing 1 (NLRP1) inflammasome to further stimulate HIF-1α expression and ultimately formed an HIF-1α-NLRP1 vicious circuit [113]. In the microglia of human AD hippocampus, the upregulation of HIF-1α and its target genes correlates with reduced coverage of Aβ plaques by microglia and an increased extent of plaque-associated neuropathology [114]. In APP knock-in mice, complement C3a receptor (C3aR)-positive microglia showed an upregulated expression of HIF-1 signaling with abnormal lipid droplet accumulation; the knockout of C3aR accompanied by reduced HIF-1α signaling reversed AD pathology with improved learning memory [115]. In astrocytes derived from 5×FAD mouse brains, compensatory mechanisms including increased HIF-1 expression was observed, which is expected to protect cells against Aβ toxicity [116]. Depending on the types of cells that express HIF-1, coupled with its multifaceted effects on handling various cellular stress, it is conceivable that HIF-1 may play very complicated roles in AD [117,118]. The potential beneficial and detrimental roles of HIF-1 pathways in AD are discussed in detail below (Figure 3).

### 3.1. The Beneficial Roles of HIF-1 in AD

#### 3.1.1. Potential Protective Mechanisms of HIF-1 in AD

Energy metabolism—Previous studies revealed a global reduction in glucose metabolism that is associated with AD patients [119]. Several genes involved in glucose transport and glycolysis, such as GLUT1/GLUT3 and phosphoglycerate kinase-1 (PGK1), are transcriptionally regulated by HIF-1 [120,121]. The expression levels of glucose transporters were downregulated in AD brains, which were correlated to abnormal tau phosphorylation and the expression of HIF-1 [110]. HIF-1 critically involved in glucose metabolism mediates neuroprotective effects against Aβ toxicity; further, both HIF-1 and those enzymes in the glycolytic pathways are crucial for neuronal survival over the frontal cortex in AD patients [122]. These studies may denote a possible mechanism underlying AD-related neurodegeneration that involves impaired brain glucose uptake/metabolism due to decreased HIF-1, resulting in deficient expression of GLUT1 and GLUT3. Aerobic glycolysis, the nonoxidative metabolism of glucose despite the presence of abundant oxygen, is a key regulator of synaptic plasticity in neurons, which requires the synthesis of macromolecules like mRNA and proteins [123]. Reduced aerobic glycolysis during aging may ruin cell survival mechanisms and fail to counteract neurodegenerative changes; notably, aerobic glycolysis is regulated at the transcriptional level by HIF-1α and peptidyl-prolyl cis/trans isomerase-1 (Pin1) [124]. Extended from these findings is that, under a critical clinical condition, those drugs capable of decreasing glucose uptake in the brains should be avoided for AD patients [125]. Besides neurodegeneration, glial activation is one of the early alterations during AD progression, possibly related to the deposition of Aβ resulting in gliosis [126,127]. In the early stage of the Alzheimer’s continuum, reactive astrogliosis is linked to higher consumption of cerebral glucose [128]. Preventing the proteolysis of HIF-1α may reverse Aβ-induced glial activation and glycolytic changes [129]. These findings again imply the crucial role of HIF-1α, which may be mediated by its transcriptional activity to maintain metabolic integrity and restore compromised energy metabolism in affected AD brains, in both neurons and glia.

Neuroprotection and neurorestoration—Hypoxia or brain hypoperfusion may contribute to AD pathogenesis [130,131]. HIF-1α, induced under such hypoxic circumstances, may play a compensatory role in coping with the diseased conditions [87]. Emerging evidence has revealed HIF-1α as a potential therapeutic target for various neurological disorders [87,132,133,134]. First, Aβ has been shown to directly induce HIF-1α expression in vitro; interestingly, low levels of Aβ protect neurons from a more severe insult by triggering HIF-1α, whereas the overexpression of HIF-1α alone is sufficient to protect neurons against Aβ toxicity [122]. Second, several downstream genes of HIF-1 are well-known to be vasoactive molecules, such as EPO, endothelial nitric oxide synthase (eNOS), and VEGF [105,117]. Notably, these proteins may also improve neuronal cell growth or survival besides their pro-angiogenic functions. For example, EPO and VEGF carry essential trophic effects during brain development and, in response to neuronal damage, exert neuroprotective or neurorestorative effects [135]. In hippocampal neurons in vitro, EPO was both necessary and sufficient to prevent Aβ-induced apoptosis in both the early and later stages of neurodegeneration, which involves expression and translocation of the p65 subunit of nuclear factor-kappaB (NF-_k_B) [136]. We have shown previously that EPO may exert neuroprotective effects against metabolic insults induced by mitochondrial inhibitor 3-nitropropionic acid (3-NP) in primary cortical neurons [137].

Neurogenesis—Sleep apnea is known to affect mental performance and may also contribute to neurodegeneration in AD [138,139,140]. Rodents under intermittent hypoxia, an animal model of sleep apnea, presented impaired spatial memory, hippocampal function, and adult neurogenesis; HIF-1α signaling activated by intermittent hypoxia in early neuroprogenitors increases the production of mature neurons upon the termination of intermittent hypoxia [141]. Chronic hypoxia also induces neurogenesis in the subgranular zone (SGZ) in the hippocampus of adult double transgenic APPswe/PS1ΔE9 mice via activation of the Wnt/β-catenin signaling pathway [142], which is HIF-1α-dependent [143]. These findings suggest that adult neurogenesis regulated by HIF-1 may represent one of its beneficial mechanisms in AD.

Counteracting oxidative stress—HIF-1 can generate the reductive equivalents of NADH/NADPH to counter oxidative stress [122]. Under hypoxic conditions, increased cytosolic anaerobic glycolysis over mitochondrial oxidative phosphorylation as a result of HIF-1 induction may also generate more pyruvate, which is an antioxidant capable of scavenging free radicals like H_2_O_2_ [144]. In another study of redox proteomics, several hippocampal proteins critical for energy metabolism, neuroplasticity, and mitogenesis/proliferation were found to be oxidatively modified in patients with mild cognitive impairment (MCI), including alpha-enolase, glutamine synthetase, pyruvate kinase M2, and Pin1; notably, the interacteome of these proteins revealed that they functionally interact with several factors including HIF-1 [145]. This study highlights the potentially antioxidative roles of HIF-1, which are expected to be beneficial in AD.

Enhancing brain circulation or angiogenesis?—Earlier it was shown that individuals suffering from severe hypoxia or ischemia are more susceptible to developing AD [146]. HIF-1α is found to be elevated in the microcirculation of AD patients [111]. In animal studies, brain sections from AD transgenic mice also showed heightened expression of HIF-1α as well as the pro-angiogenic proteins including angiopoietin-2 (Ang-2) and matrix metalloproteinase-2 (MMP2) in the brain vasculature [112]. Conceivably, it seems reasonable to predict that HIF-1 activation may exert beneficial effects against AD by enhancing angiogenesis or augmenting cerebral blood flow. Despite these lines of correlative evidence in clinics and animal models in vivo, however, direct causative evidence supporting this beneficial mechanism of HIF-1α in AD is still lacking in the literature. On the contrary, the inhibition of HIF-1α expression was shown to mediate the beneficial effects of schisandrin B, an active component derived from the Chinese herb Wuweizi, in the rat bilateral common carotid artery occlusion (BCCAO) as a vascular dementia model [147]. Therefore, whether enhancing HIF-1 activity may have positive impacts on AD by augmenting brain circulation or enhancing angiogenesis requires further investigation.

#### 3.1.2. Multiple Chemical Reagents and Proteins Exert Beneficial Effects on AD via Induction of HIF-1

Earlier it was demonstrated that iron is required for Aβ toxicity and iron chelators have neuroprotective effects [148]. This may in part due to the fact that aberrant iron metabolism, especially the ferrous iron (Fe^2+^) in the cytoplasmic labile iron pool, produces superoxide anions (O_2_·) and a strong ROS hydroxyl radical (OH·) through a Fenton reaction with H_2_O_2_.; the resultant ROS cause oxidative damage to lipids, proteins, and DNA, thus leading to cell death and affecting Aβ misfolding along with plaque aggregation [149]. Furthermore, in the presence of Fe^2+^ and/or lipoxygenases, the polyunsaturated fatty acids in the cell membrane catalyze lipid peroxidation to trigger ferroptosis, an iron-specific programmed cell death, which is closely related to the occurrence, development, and prognosis of AD [150]. Transferrin receptor 1 (TfR1) is a key player in the regulation of the brain distribution of iron. One recent study suggested that, in AD under hypoxia and other non-hypoxic stimuli, such as oxidative stress and inflammation, TfR1 upregulation may be associated with HIF-1 activation causing iron dyshomeostasis in the brain, which ultimately contributes to AD pathology [151]. In this regard, iron chelation may be considered a therapeutic option for AD [152]. Consistently, an iron chelator M30 capable of upregulating HIF-1α with heightened expression of its downstream target genes, including enolase-1, EPO, p21, tyrosine hydroxylase, VEGF, and insulin signaling pathway, in cortical neurons and APP/PS1 AD mice, may have anti-Alzheimer characters [153,154] (Table 1). In APP/PS1 transgenic mice, intranasal delivery of the iron chelator DFO can upregulate the p38/HIF-1α pathway and lessen synaptic loss in the brain [155]. Intranasal DFO treatment also improves memory in healthy mice that is accompanied by reduced GSK-3β activity and intensified HIF-1α activity [156]. In primary cultures of rat and mouse astrocytes, DFO stabilizes HIF-1α to inhibit both PHDs and the proteasome, thereby mitigating Aβ-induced glial activation with enhanced pentose shunt to generate more NADPH for restricting ROS accumulation [129]. We have also shown before that HIF-1 induction with cobalt chloride or DFO can protect rat C6 glioma cells against metabolic insults induced by the irreversible mitochondrial inhibitor 3-NP [157]. These results together denote potential beneficial effects of iron chelators, such as M30 and DFO, through HIF-1α stabilization in AD.

In addition to iron chelators, several chemical compounds or proteins exert beneficial effects against AD via the induction or stabilization of HIF-1α. Recently, it was shown that gut dysbiosis, or disturbance in the gut microbiota, can lead to inflammation and is associated with the pathogenesis of various diseases including AD [158,159]. The term “brain–gut axis” denotes crosstalk between the brain and the gut that involves multiple overlapping pathways, which comprise the autonomic functions, immune systems, neuroendocrine, neuro-modulatory molecules, and bacterial metabolites [160]. It is imperative to understand the mechanisms inherent in the microbiota–gut–brain axis so that microbe-based intervention and therapeutic strategies may be developed for neurodegenerative diseases [161]. One previous study employing 3×Tg-AD mice revealed that chronic supplementation with SLAB51, a multi-strain probiotic formulation, can augment the cerebral expression of HIF-1α, possibly by reducing the level of PHD2 critical for HIF-1α degradation; SLAB51 also decreases the expression of inducible nitric oxide synthase (iNOS) in the brain, with a reduction in NO levels in the plasma of AD mice [162]. This study adds an additional mechanism for probiotics to reduce oxidative stress and inflammation in AD models through the regulation of HIF-1α expression. The lactoferrin present in milk is an iron-binding glycoprotein with pleiotropic functions. Recently, lactoferrin has been suggested as a neuroprotective agent because the intranasal delivery of lactoferrin into AD mice can augment α-secretase-dependent APP processing through the ERK1/2-CREB and HIF-1α pathways [163]. The beneficial effects of lactoferrin in reducing Aβ aggregation with improved spatial learning ability were also revealed. As a highly accessible nutrient supplement, further clinical studies on the advantageous effects of lactoferrin for AD are warranted. Neuroglobin is a hypoxia-inducible protein with protective effects in animal models of AD, stroke, and related nervous system disorders [164]. It was demonstrated that the knockdown and overexpression of HIF-1α, respectively, reduced and increased neuroglobin levels, consistent with a causal relationship between HIF-1 and neuroglobin induction [165]. This finding adds one more mechanism for the beneficial effect of HIF-1α expression and neuroglobin with potential therapeutic values for AD. α-Lipoic acid can maintain brain glucose metabolism through the BDNF/TrkB/HIF-1α signaling pathway in P301S mice, a transgenic mouse model for tauopathy; chronic α-lipoic acid administration into the P301S mouse brains elevated the expression of GLUT3, GLUT4, VEGF, and HO-1 at mRNA or protein levels that together increased glucose availability [166]. Finally, recent epidemiological studies and experimental evidence suggested that coffee consumption lowered the risk of cognitive disorders including AD [167,168]. Coffee was found to induce VEGF expression in human neuroblastoma SH-SY5Y cells through the activation of HIF-1α, which was related to the inhibition of prolyl hydroxylation independent of caffeine or caffeic acid [169]. A previous study revealed that a single nucleotide polymorphism (SNP) rs1868402 in the *PPP3R1* gene encoding protein phosphatase 3 regulatory subunit B (PPP3R1) is significantly associated with the rapid progression of AD [170]. A low level of PPP3R1 proteins showed a strong correlation with AD and may serve as a potential biomarker for predicting and preventing AD for future development of personalized medicine; notably, PPP3R1 was found to be involved in the HIF-1 pathway [171]. Not surprisingly, the direct enhancement of HIF-1 activity or its specific downstream target genes is expected to also provide protective effects against AD. For example, viral vectors expressing HIF-1α may inhibit hippocampal neuronal apoptosis induced by Aβs both in vivo and in vitro [172]. Apolipoprotein E4 (ApoE4), the most common genetic risk factor for sporadic AD, is associated with more evident neurodegeneration and vascular impairments [173,174]. ApoE4-driven brain pathology revealed a specific decrease in both VEGF receptor-2 and HIF-1α. Using VEGF-expressing adeno-associated virus (AAV-VEGF) driven by the GFAP promoter that allows VEGF expression exclusively in astrocytes, it was demonstrated that the AAV-VEGF may reverse the ApoE4-related Aβ42 aggregation and accumulation of hyperphosphorylated tau proteins [174]. This evidence indicates the critical role of HIF-1 in AD patients with ApoE4. Further study is warranted to manipulate the HIF-1 expression in these subgroups of AD to examine its potential clinical outcomes. These findings together support the potential use of gene therapy for the treatment of neurodegenerative diseases including AD.

**Table 1:** List of examples with beneficial roles of HIF-1 in AD-related studies.

Chemical Compound, Drug, Nutrient, Protein	Mechanisms	Study Model	Reference
M30, an iron chelator	upregulated HIF-1α and its target genes: enolase-1, erythropoietin, p21, tyrosine hydroxylase, VEGF, Glut-1	in vivo: APP/PS1 double Tg mice in vitro: rat primary cortical neurons	[153,154]
DFO, an iron chelator	* upregulated the p38/HIF-1α pathway and lessened synaptic loss in the brain ** reduced GSK-3β activity and intensified HIF-1α activity *** increased HIF-1α, inhibited PHD2 and the proteasome, reduced glial activation, produced more NADPH to limit ROS accumulation	in vivo: APP/PS1 Tg mice in vivo: healthy mice in vitro: mouse and rat astrocytes	[155] [156] [129]
SLAB51, a multi-strain probiotic formulation	increased HIF-1α, decreased PHD2, iNOS in brain, and NO levels in plasma	in vivo: 3 × Tg-AD and wild-type mice	[162]
Lactoferrin, a nutrient derived from milk	augmented α-secretase-dependent APP processing through the ERK1/2-CREB and HIF-1α pathways, reduced Aβ aggregation	in vivo: APP/PS1 double Tg mice	[163]
Neuroglobin, a hypoxia-inducible protein with cytoprotective effects	shRNA-mediated knockdown and lentiviral vector-mediated overexpression of HIF-1α	in vitro: HN33 neural cell line	[165]
α-Lipoic acid	increased GLUT3, GLUT4, VEGF, and HO-1 expression with enhanced glucose availability through BDNF/TrkB/HIF-1α signaling	in vivo: tauopathy model, P301S mice	[166]
Coffee	inhibited prolyl hydroxylation to activate HIF-1α and induced VEGF expression	in vitro: SH-SY5Y human neuroblastoma cell line	[169]
Viral vector expressing HIF-1α	inhibited hippocampal neuronal apoptosis induced by Aβ protein	in vitro: primary culture of hippocampal neurons in vivo: Sprague-Dawley rats	[172]

APP/PS1: amyloid precursor protein/presenilin-1, DFO: desferoxamine, GLUT-1: glucose transporter-1, HO-1: heme oxygenase-1, iNOS: inducible nitric oxide synthase, NADPH: nicotinamide adenine dinucleotide phosphate, PHD2: prolyl hydroxylase 2. * denotes Ref [155]; ** denotes Ref [156]; *** denotes Ref [129].

### 3.2. The Detrimental Roles of HIF-1 in AD

#### 3.2.1. Potential Detrimental Mechanisms of HIF-1 in AD

Although some experimental evidence supports the beneficial effects of the HIF-1 pathway in AD, unfavorable impacts of HIF-1 activation in AD have also been reported. For example, HIF-1α can affect APP processing by regulating the activities of α-, β-, and γ-secretase, thereby increasing the generation of Aβ or decreasing the secretion of sAPPα. Hypoxia, and possibly HIF-1α, may also modulate the expression or activities of Aβ-degrading enzymes like neprilysin (NEP), endothelin-converting enzyme (ECE)-1, and insulin-degrading enzyme (IDE). HIF-1α may also modulate microglial activation to aggravate the severity of neuroinflammation, which is expected to expedite AD pathogenesis [175]. Other possible detrimental effects of HIF-1 in AD include impairing the integrity of the blood–brain barrier (BBB) and compromising the microvasculature. For example, in rat brain capillary endothelial cells (RBE4), oxygen–glucose deprivation (OGD), as an in vitro model of hypoxia/ischemia, elicits Aβ42 production through HIF-1-mediated BACE1 upregulation [176]. Thus, ischemic events may directly contribute to the enhancement of the amyloidogenic metabolism in brain capillary endothelial cells, leading to intracellular deposition of Aβ42, impaired Aβ clearance, and AD-related BBB dysfunctions.

APP processing by BACE1/β-secretase—Previous studies suggested that hypoxia may contribute to AD pathogenesis by overexpressing APP and increasing Aβ formation [177,178]. Hypoperfusion caused by focal ischemia induces APP expression at mRNA levels [179]. Prolonged hypoxia increased production of Aβ that selectively increased the expression of L-type Ca^2+^ channels, which is considered detrimental in AD; hypoxia also promoted physical association of Aβ with the α1C subunit of the L-type Ca^2+^ channel that likely contribute to the Ca^2+^ dyshomeostasis of AD [180,181]. These earlier findings collectively revealed the link between hypoxia/ischemia and APP expression/processing that may contribute to AD pathogenesis. The direct involvements of HIF-1 in APP processing were later confirmed. For example, hypoxia increases BACE1 gene transcription at mRNA levels through the induction of HIF-1 with resultant increased β-secretase activity and Aβ production; notably, gel shift assays reveal HIF-1 binding to the HREs in the BACE1 promoter; indeed, the overexpression of HIF-1α is sufficient to increase BACE1 at both the mRNA and protein levels, whereas the downregulation of HIF-1α reduces the level of BACE1 [182]. A further study revealed that hypoxia triggers BACE1 expression through a biphasic mechanism; the early stage is mediated by the mitochondrial electron transport chain and the later stage is caused by the activation of HIF-1 and oxidative stress [183].

APP processing by α-secretase?—Besides enhancing BACE1 expression along with increased β-secretase activity, chronic hypoxia decreases the protein expression of a disintegrin and metalloproteinase 10 (ADAM10), which is considered an α-secretase mediating the non-amyloidogenic processing of APP, in the neuronal SH-SY5Y cells [184,185], along with the suppressed secretion of sAPPα [184]. However, the direct involvements of HIF-1α were not reported in these two studies.

PS1/2 and γ-secretase?—The γ-secretase is responsible for further digesting the α-CTF (C83) and β-CTF (C99) following the nonamyloidogenic and amyloidogenic cleavage of APP by α- and β-secretase, respectively. Being a high-molecular-weight complex consisting of four components, namely presenilin-1/2 (PS1/2), nicastrin (NCT), anterior pharynx-defective-1 (APH-1), and presenilin enhancer-2 (PEN-2), PS1/2 is the catalytic subunit in the γ-secretase complex [186]. Brain hypoxia induced by cerebral hypoperfusion or breathing 10% O_2_ triggered HIF-1α binding to γ-secretase to enhance its biological activity without affecting the expression level of individual subunits; further, the expression of full-length HIF-1α in primary neurons increased BACE1 expression and γ-secretase activity in a transcriptional and non-transcriptional manner, respectively, for Aβ production under hypoxic conditions [187]. In another earlier study, the activation of HIF-1 by short-term NiCl_2_ treatments, a condition known as chemical hypoxia, substantially increases APH-1A expression at both mRNA and protein levels without affecting APP or the other three components of the γ-secretase complex; importantly, NiCl_2_ treatments also lead to an increased secretion of Aβ and decreased formation of APP CTFs, indicative of elevated γ-secretase activity [188]. Importantly, loss-of-function mutations in PS1/2 or mutations in PS1 associated with familial AD (FAD) may also affect HIF-1 induction. PS1/2 γ-secretase-mediated cleavage of the APP generates AICD, which functions as a transcriptional activator to induce *Hif1a* gene expression; on the other hand, PS1/2 itself, in a γ-secretase-independent manner, increases PHD2 activity to promote HIF-1α degradation in vitro [189]. Using fibroblasts lacking PS1, the induction of HIF-1α was impaired in response to cobalt chloride, which is known to stabilize HIF-1α under normoxia, or by insulin [190]. The lentivirus-mediated expression of human PS1 in part rescued the responsiveness of PS1-/- fibroblasts to cobalt chloride induction; however, HIF-1α induction did not require γ-secretase activity. Interestingly, PS1 and HIF-1α appeared to physically interact with each other, suggesting that PS1 may protect HIF-1α from degradation [190]. In fibroblasts harboring the M146V PS1 FAD mutation on a mouse PS1-null background, the metabolic induction of HIF-1α by insulin was impaired, but not by cobalt chloride [190]. These findings indicate complex mechanisms whereby PS1/γ-secretase, both wild-type and those containing FAD mutations, modulate the induction of HIF-1α.

Aβ degradation?—In addition to regulating APP expression and processing by secretase activities, HIF-1 is also involved in Aβ degradation. Neprilysin (neutral endopeptidase, NEP) is a zinc-dependent metalloprotease responsible for Aβ degradation [191]. In prostate cancer cell lines and human umbilical vascular endothelial (HUVEC) cells, hypoxia inhibits the expression of NEP at mRNA levels; out of the three putative HREs upstream of the NEP promoter, two demonstrate a specific reduction, rather than an increase, in cobalt-induced HIF-1 binding in gel shift assays [192], suggesting a negative impact on NEP expression on the binding of HIF-1 to these HREs. In contrast to these findings, one study conducted in neuroblastoma cells reported that HIF-1α mediates the upregulation of NEP through HIF-1α binding to histone deacetylase (HDAC)-1, thereby reducing the association of HDAC-1 with the NEP promoter, with a resultant activation of its transcription in a de-repression manner [193]. The causes underlying these contradictory results regarding the HIF-1-mediated regulation of NEP require further investigation, but several contributing factors may be considered, including the different cell types (HUVEC versus N2a neuroblastoma cells) as well as how HIF-1α was induced (cobalt chloride versus hypoxia under low-oxygen tension). Another Aβ-degrading enzyme, ECE-1, has also been proposed to be an HIF-1 target gene in endothelial cells [194]. Hypoxia downregulated the expression of IDE, another peptidase known to degrade Aβ, in U87 glioma cells [195], but the potential roles of HIF-1α remain unclear. Taken together, understanding whether HIF-1 directly regulates the expression or activities of these Aβ-degrading enzymes and their potential impacts on AD pathogenesis requires more investigation.

Tau hyperphosphorylation?—Depending on the experimental model systems, how HIF-1α may affect tau hyperphosphorylation remains controversial. In a clinical study, two major brain glucose transporters, GLUT1 and GLUT3, responsible for glucose uptake into neurons, were decreased in AD brains; notably, this decrease correlated to the hyperphosphorylation of tau and downregulation of HIF-1α, but not HIF-1β [110]. Since HIF-1 is a major regulator of GLUT1 and GLUT3, these studies provide a piece of correlative evidence linking tau hyperphosphorylation to HIF-1α downregulation in AD. Accordingly, HIF-1α has also been shown to suppress tau phosphorylation. For example, one recent study reported that T-2 toxin, a mycotoxin that may potentially lead to the progression of AD, stimulates HIF-1α expression, along with induction of APP and hyperphosphorylation of tau proteins in microglial BV2 cells; intriguingly, further studies revealed that the T2-induced HIF-1α actually functions as a “brake” to negatively regulate APP induction and tau phosphorylation, suggesting a protective effect of HIF-1α in microglial cells challenged with T-2 toxin, in part by suppressing tau hyperphosphorylation [196]. In contrast, however, HIF-1α has also been shown to indirectly contribute to tau phosphorylation. As a risk factor for AD, chronic hypoxia upregulates HIF-1α, which decreases the activity of protein phosphatase-2A (PP2A), thereby mediating tau hyperphosphorylation with resultant cognitive dysfunction [197]. Cobalt, as an environmental toxicant that is also known to stabilize HIF-1α, severely induces Aβ deposition, tau hyperphosphorylation, and dysregulated autophagy in the hippocampus and cortex of mice; importantly, HIF-1α knockdown by siRNA effectively attenuated the increased tau phosphorylation at Thr181 induced by cobalt [198], suggesting that HIF-1α is responsible for cobalt-dependent tau phosphorylation. Finally, HIF-1α may also mediate the beneficial effects of small-molecule compounds without notably affecting tau phosphorylation. The iron chelator DFO, known to stabilize HIF-1α, improves memory in healthy C57 mice [156] and also exerts protective effects in P301L mice [199]. These transgenic mice overexpress the human 4R/2N tau isoform bearing the P301L mutation under the control of the neuron-specific murine Thy1 promoter with an accumulation of hyperphosphorylated tau, which serves as a transgenic model for AD and frontotemporal dementia (FTD). It was found that P301L mice have significantly less HIF-1α with more severe total protein oxidation than wild-type controls, whereas the intranasal delivery of DFO significantly reversed these differences; however, no significant decrease in phosphorylated tau was observed in the brains of these DFO-treated mice, at least for the brain regions examined [199]. Depending on the experimental model systems, therefore, HIF-1α may enhance, suppress, or have no direct effects on the phosphorylation status of tau proteins. Obviously more investigation is required to establish the definite roles of HIF-1 in tau hyperphosphorylation.

Microglia and neuroinflammation?—HIF-1 may affect microglia in different aspects. Using methoxy-X04 (X04), a brain-penetrant fluorescent Aβ probe, to isolate plaque-containing (X04^+^) and non-containing (X04^-^) microglia from 5×FAD mouse brains for transcriptome analysis, it was found that HIF-1α and its downstream target genes are involved in the enhanced phagocytosis of synaptic components around plaques and, through a feed-forward loop, ultimately augment Aβ phagocytosis in microglia [200]. The rapid pruning of damaged synapses near dystrophic neurites around plaques and the enhancement of Aβ phagocytosis may be considered protective and, in this regard, HIF-1 likely plays a beneficial role in microglia-dependent phagocytosis in AD. In contrast, however, excessive HIF-1 activity may have detrimental effects on microglia in AD. For example, the prolonged activation of HIF-1α results in cell cycle arrest along with the impaired proliferation of microglia; under in vivo condition, the overstabilization of HIF-1α reduces the proliferation and clustering of plaque-associated microglia along with increased Aβ neuropathology [114]. It has been demonstrated that hypoxia may also induce autophagic cell death and inflammatory responses through HIF-1α in microglia [201], although its potential impact on AD requires more investigation. In addition to affecting phagocytosis, cell proliferation, clustering around Aβ plaques, and possibly autophagic cell death, the HIF-1α pathway plays a pivotal role in the metabolic program and immune responses in microglia. The exposure of microglia to Aβ triggers acute inflammation along with metabolic reprogramming from oxidative phosphorylation to glycolysis, which depends on the mTOR-HIF-1α pathway; however, chronic exposure to Aβ leads to an overall defective energy metabolism with diminished immune responses, including cytokine secretion and phagocytosis [202]. Overall, conditions with prolonged or overwhelming stress like severe or chronic hypoxia may alter the beneficial roles of HIF-1 such that it is converted into a potential activator of cell death in AD [133].

Brain hypoperfusion and impaired vascular functions—As mentioned above, microvessels isolated from the brains of AD patients [111] as well as AD transgenic mice [112] express a number of angiogenic proteins along with elevated HIF-1α expression. However, direct evidence supporting the beneficial mechanism of enhancing cerebral blood flow or microcirculation by HIF-1α in AD models is still lacking. On the contrary, it has been reported that chronic cerebral hypoperfusion commonly observed in AD patients may induce HIF-1α to cause BBB damage and ultimately impair Aβ clearance [203]. Another recent study revealed that chronic hypoxia capable of activating HIF-1 is detected in AD brains and retinas, especially in microvascular endothelial cells, which leads consequently to the formation of NLRP1 inflammasome and upregulates the signaling cascades of “adaptor molecule apoptosis-associated speck-like protein containing a CARD (ASC)-caspase-1- IL-1β”. In turn, NLRP1 can reciprocally stimulate HIF-1α expression to reinforce the HIF-1α-NLRP1 circuit, which may further destroy the vascular system and thus suggest a detrimental role of HIF-1α in microvascular structures in AD [113]. These negative impacts of HIF-1α in the microvasculature of AD, along with the aforementioned HIF-1 effects on APP processing and Aβ production/degradation, may further impair vascular function in AD and compromise BBB function, leading to impaired Aβ clearance [204].

Neuronal cell cycle reentry—Emerging evidence indicates that aberrant cell cycle reentry and subsequent apoptosis in fully differentiated neurons occur during the advancement of AD or other neurodegenerative diseases [205,206,207]. We have shown before that the signal transduction pathways involving the inhibitor of DNA-binding/differentiation protein-1 (Id1), HIF-1α, cyclin-dependent kinases-5 (CDK5), sonic hedgehog, and protein kinase C-delta (PKCδ) may contribute to Aβ-induced cell cycle reentry and neurotoxicity in fully differentiated postmitotic neurons [15,16,206,208,209,210], adding another piece of evidence supporting the detrimental role of HIF-1α in AD under selected circumstances.

#### 3.2.2. Proteins and Chemical Reagents Exert Beneficial Effects in AD via Inhibition of HIF-1

Recently, the relationship between dementia and diabetes mellitus has inspired researchers to explore new therapeutic potential from glucose regulators for devastating neurodegenerative diseases like AD or PD [211,212,213]. Fibroblast growth factor 21 (FGF21), a member of the FGF family, is a liver-secreted peptide hormone encoded by the *fgf21* gene [214]. Positively implicated in the regulation of energy, glucose, and lipid metabolism, FGF21 is an appealing and promising therapeutic target for DM [215]. However, FGF21 may also present miscellaneous influences on the central nervous system [216,217]. For example, FGF21 lessened tau hyperphosphorylation and oxidative stress in cellular and rat models of AD; its beneficial effects involved the inhibition of the PP2A/MAPKs/HIF-1α pathway [216] (Table 2). Heparin-binding EGF-like growth factor (HB-EGF), an EGF family member widely distributed in neurons and glia, can be induced by hypoxia and/or ischemia, which contributes to chronic cerebral hypoperfusion (CCH)-mediated Aβ accumulation [218]. An HB-EGF-dependent increase in HIF-1α expression can activate matrix metalloprotease-9 (MMP9) to cause BBB disintegration [203]. Neuregulin 1 (NRG1), which belongs to the epidermal growth factor family, is thought to play a role in synaptic plasticity [219,220]. Cobalt chloride, which is known to stabilize HIF-1α, induced cytotoxicity along with the increased expression of HIF-1α and p53 in SH-SY5Y cells; notably, pretreatment with NRG1 inhibited the accumulation of HIF-1α, decreased p53 stability, and significantly mitigated cell death in the SH-SY5Y cells exposed to cobalt chloride [221]. These studies revealed the detrimental effects of HIF-1 by various protein mediators in Aβ-induced neurotoxicity or AD animal models, both in vivo and in vitro.

In addition to protein mediators, various cellular environments or growth conditions may affect HIF-1α induction and AD pathology. Under high-glucose condition, BACE1 is upregulated with increased Aβ production through HIF-1α activation and decreased LXRα expression via the JNK pathway in a ROS-dependent manner in SK-N-MC, a neuroblastoma cell line [222]. Thiamine (Vitamin B1) insufficiencies (TI) have been associated with AD-like neuropathology. Using the HT22 neuronal hippocampal cell line, it was demonstrated that TI-induced HIF-1α triggers amyloidogenesis through the transcriptional expression of BACE1 along with increased β-secretase activity (BACE1); furthermore, TI also induces the expression of pro-apoptotic protein BNIP3 via HIF-1α and, correspondingly, neurotoxicity caused by TI conditions can be significantly reduced with the knockdown of HIF-1α and BNIP3 [223]. This study suggests that, under selected circumstances, the induction of HIF-1α may also trigger the expression of pro-apoptotic genes that contribute to neuronal death.

Several compounds may also exert beneficial effects through the inhibition of the HIF-1 pathway. Ginsenoside Rg1, one of the active ingredients in *Panax ginseng*, can ameliorate declined cognitive function and reduce cerebral Aβ levels [224,225]. It was demonstrated that Rg1 reduced Aβ-induced mitochondria-dependent apoptosis in human endothelial cells, which involved lessened HIF-1α expression accompanied by diminished ROS production and protein nitrotyrosination [226]. Salidroside, a glucoside of tyrosol found in the plant *Rhodiola rosea*, has been reported to mitigate hypoxia-induced abnormal APP processing in SH-SY5Y neuronal cells [227]. Salidroside pretreatment significantly decreased the expression of BACE1 at both mRNA and protein levels along with inhibited β-secretase activity, thus attenuating Aβ generation induced by hypoxia without affecting γ-secretase activity. Salidroside also promoted the secretion of sAPPα in hypoxic condition without affecting APP, ADAM10, and ADAM17; the latter two are considered α-secretases, suggesting that heightened sAPPα secretion may be the consequences of decreased β-secretase activity. Notably, under hypoxic conditions, salidroside pretreatment reduced the protein level of HIF-1α, suggesting that salidroside may attenuate a hypoxia-induced increase in BACE1, possibly through downregulating the HIF-1α protein level [227]. A high-fat diet stimulates amyloidogenic pathways, which is critical for the pathogenesis of AD [228]. Palmitic acid-BSA (PA-BSA) treatment stimulates BACE1 expression in astrocytes with elevated oxidative stress [229,230]. It was found that PA-BSA-induced Aβ production involved the activation of Akt/mTOR/HIF-1α and Akt/NF-κB pathways to stimulate the expression of APP and BACE1; silencing both genes correspondingly attenuated the production of Aβ [231]. Neurotropin^®^ (NTP), a well-known drug for chronic pain, was found to stimulate BDNF expression in SH-SY5Y cells and restore the declined expression of BDNF in the hippocampus [232]. It was reported that Neurotropin^®^ possesses a neuroprotective character by lessening Aβ-induced oxidative damage and alleviating Aβ deposition in the hippocampus through the downregulation of the HIF-1α/MAPK signaling cascade [233]. By transferring mitochondria from a living sporadic AD [205] patient into mitochondrial DNA (mtDNA)-free SH-SY5Y cells, mitochondrial transgenic neuronal cells, or cybrids, can be produced [234,235]. Using the cybrids of SAD and age-matched controls, it was demonstrated that, under hypoxic conditions with different dosages of simvastatin, the extents of HIF-1α and BACE induction may vary in these cybrids. In the low-dose simvastatin group, the reduced expression of HIF-1α and BACE1 was observed in the SAD cybrids as compared to the controls. In the high-dose simvastatin group, however, the heightened expression of HIF-1α and BACE1 was observed in both the SAD and the control cybrids. These results denote the potential usefulness of low-dose simvastatin treatment in attenuating the expression of HIF-1α and BACE to lower Aβ production [236]. In human neuroblastoma SH-SY5Y cells under the in vitro ischemic condition of OGD and OGD with reoxygenation (OGD/R), HIF-1α was highly upregulated at both the mRNA and protein levels, which were downregulated by melatonin; consistently, the heightened BACE1 mRNA and proteins under such ischemic condition were suppressed by melatonin [237]. However, a direct causal relationship between melatonin-induced HIF-1α and BACE1 under ischemic conditions was not established in this study.

**Table 2:** List of examples with detrimental roles of HIF-1 in AD-related studies.

Chemical Compound, Drug, Nutrient, Protein	Mechanisms	Study Model	Reference
Fibroblast growth factor 21 (FGF21)	inhibition of PP2A/MAPKs/HIF-1α pathway triggered by Aβ25-35	in vitro: SH-SY5Y human neuroblastoma cell line in vivo: adult male Wistar rats	[216]
HB-EGF	increased HIF-1α expression can activate MMP9 to cause BBB disintegration	in vivo: BCCAO mouse model, CCH manifests AD neuropathology	[203]
Neuregulin 1	inhibited CoCl_2_-induced accumulation of HIF-1α and p53 stability to attenuate cell death	in vitro: SH-SY5Y human neuroblastoma cell line	[221]
High glucose condition	upregulated BACE1-mediated Aβ production through HIF-1α activation, decreased LXRα expression via the JNK pathway in a ROS-dependent manner	in vitro: SK-N-MC neuroblastoma cell line in vivo: homozygous ZDF and ZLC rats	[222]
Thiamine insufficiency (TI)	triggered HIF-1-mediated amyloidogenesis through transcriptional expression of BACE1 and increased activity of β-secretase- induced HIF-1-dependent expression of pro-apoptotic protein BNIP3	in vitro: HT22 hippocampal neuronal cell line	[223]
Ginsenoside Rg1, active ingredients of *Panax ginseng*	reduced the Aβ-induced mitochondrial apoptosis, lessened HIF-1α expression, decreased RNS and protein nitrotyrosination	in vitro: human endothelial cells	[226]
Salidroside, a glucoside of tyrosol found in the plant Rhodiola rosea	reduced protein level of HIF-1α under hypoxia; decreased expression of BACE1 and inhibited β-secretase activity, thus enhancing sAPPα secretion and attenuating Aβ generation induced by hypoxia	in vitro: SH-SY5Y human neuroblastoma cell line	[227]
PA-BSA	induced expression of APP and BACE1, accelerated Aβ production via Akt/mTOR/HIF-1α and Akt/NF-κB pathways	in vitro: SK-N-MC neuroblastoma cell line	[231]
NTP, a non-protein extract of inflamed rabbit skin inoculated with vaccinia virus clinically used for the treatment of neuropathic pain	lessened Aβ-induced oxidative damage, improved Aβ deposition in hippocampus through regulating HIF-1α/MAPK signaling pathway	in vitro: HT22 hippocampal cells in vivo: APP/PS1 Tg mice	[233]
Simvastatin, in low dosage	reduced HIF-1α and BACE1 expression	in vitro: sporadic AD and age-matched control neuronal mitochondrial cybrid	[236]
Melatonin	suppressed HIF-1α and BACE1 expression under the in vitro ischemic condition induced by OGD and OGD/R	in vitro: SH-SY5Y human neuroblastoma cell line	[237]

BCCAO, bilateral common carotid artery occlusion; BNIP3, BCL2/adenovirus E1B 19-kDa protein-interacting protein; CCH, chronic cerebral hypoperfusion; HB-EGF, heparin-binding EGF-like growth factor; NTP, Neurotropin^®^; OGD and OGD/R, oxygen–glucose deprivation and OGD with reoxygenation; PA-BSA, palmitic acid-bovine serum albumin; RNS, reactive nitrogen species; TI, thiamine insufficiency; ZDF and ZLC rats, Zucker diabetic fatty and Zucker lean control fatty rat.

## 4. Conclusions and Future Perspectives

AD is the most prevalent neurodegenerative disease in the aging population with progressive memory impairment, deteriorated cognition, and behavioral dysfunctions. At present, there is no effective treatment for this devastating disease. Any means to delay or lessen the consequences of AD may therefore represent a significant improvement for patients, their families, and societies. HIF-1α, a master regulator for gene expression under hypoxic conditions, can increase cell proliferation, accelerate new blood vessel formation, and regulate glucose and energy metabolisms to cope with a stressful cellular milieu such as hypoxia. HIF-1α is also known to be activated under oxidative stress, neuroinflammation, and cerebral hypoperfusion, the conditions often accompanied by AD. Increasing the transcriptional activity of HIF-1α can enhance angiogenesis and erythropoiesis, trigger anti-apoptotic cascades, and regulate autophagy, and thus represent a potential therapeutic scheme in the treatment of neurodegenerative disorders including AD. In contrast, however, ample evidence also indicates that HIF-1α inhibition might exert a beneficial effect on AD. Additionally, the effect of the HIF signaling greatly depends on the extent of hypoxic magnitude, severity of the stress, and the cell types that express HIF-1α. Since HIF-1 signaling could be either beneficial or detrimental, there is a pressing need to design highly selective modulators, either activators or inhibitors, for manipulating the HIF-1 transduction pathway as well as effective means of the targeted delivery of these HIF-1 modulators into proper brain regions or even cell types for AD therapy.

## Data Availability

Not applicable.
